# Decreased wheat production in the USA from climate change driven by yield losses rather than crop abandonment

**DOI:** 10.1371/journal.pone.0252067

**Published:** 2021-06-17

**Authors:** Oladipo S. Obembe, Nathan P. Hendricks, Jesse Tack

**Affiliations:** 1 College of Agriculture, Arkansas State University, Jonesboro, Arkansas, United States of America; 2 Department of Agricultural Economics, Kansas State University, Manhattan, Kansas, United States of America; Instituto Agricultura Sostenible, SPAIN

## Abstract

An increase in global average surface temperature over the 21st century will affect food production. There is still uncertainty if the source of the production losses caused by climate change could be driven either by lower yield or reduced area harvested. We use county-level production data on winter wheat coupled with fine-scale weather outcomes between 1981-2007 to examine the impact of climate change on winter wheat production in Kansas. We decompose the total impact of weather variables through both the yield and harvested acreage channels. We find that an insignificant portion—both in terms of magnitude and statistical significance—of the production losses are due to reduced harvested acres (i.e., crop abandonment). The proportion harvested only account for 14.88% and 21.71% of the total damages under RCPs 4.5 and 8.5 and neither effect is statistically significant. An implication of this result implies that studies that only examine climate impacts on harvested yields are not significantly underestimating the climate change impacts on production.

## Introduction

Changing climatic conditions are becoming one of the major challenges facing agricultural production globally as demand for staple foods increases. Despite the advancements in agricultural production through improved technology [1], risk due to climate change has also increased [2, 3]. Wheat is one of the most important staple foods consumed globally, with the USA producing 8% of the world’s total production [3–5]. While global wheat demand is expected to increase by 26% by mid-century [6], wheat production is projected to be negatively impacted by climate change [7–9].

There are two primary sources of variation in crop production: yield and harvested acres. There is a vast literature that uses statistical models to estimate the impact of weather shocks on yield, where yield is measured as total production divided by harvested acres [10–17]. These papers implicitly assume that harvested acres do not change with climate change. According to Cui [18] and Ramankutty [19], using yield calculated from harvested acres as the sole source of production variability could significantly underestimate the impact of climate change on total production. This could be especially important for overwintering crops that are typically exposed to a wider range of weather shocks during the growing season. Therefore, we investigate the impacts of weather shocks on winter wheat production in Kansas by accounting for changes in harvested acres overtime.

Wheat ranks third among U.S. field crops in planted acreage, production, and gross farm receipts, behind corn and soybeans [4]. Winter wheat represents 65–76% of the wheat production in the USA, and Kansas is the top producing state—producing between 13–21% of the total winter wheat production between 2000 and 2019. While Kansas ranks first in wheat planted acreage and production, it also ranks as one of the top states in the number of acres planted but not harvested. Only Oklahoma and Texas have larger proportions not harvested—more than 30% of planted acres in these states on average—but the lack of harvested acres in these states is likely driven by grazing rather than abandonment due to poor crop conditions.

Our objective is to estimate the responsiveness of winter wheat production in Kansas to weather shocks through changes in harvested yield (total production divided by harvested area) and the proportion of the planted area harvested using econometric models. We leverage estimates from the econometric models to simulate the impacts from mid-century (2034–2065) climate change projections under two Representative Concentration Pathways (RCPs), 4.5 and 8.5. Our results indicate that production is projected to decrease by 16.96% and 31.33% under RCPs 4.5 and 8.5, respectively. We find that freezing temperatures in the fall and extreme heat in the spring are the major drivers of yield reduction, while freezing conditions and extreme heat in the spring are associated with harvested acreage reductions.

A key distinction of our paper is that we estimate how weather impacts the proportion of acres harvested—or equivalently, the proportion of acres not abandoned. Crop abandonment occurs when an adverse weather shock reduces the yield below the point where the value of production equals the cost of harvesting. We find that decreases in the proportion of area harvested only account for 14.88% and 21.71% of the total damages under RCPs 4.5 and 8.5 and neither effect is statistically significant. Therefore, using yield impacts alone to measure the climate change impact on production does not significantly underestimate the total impact of climate change on production.

Our work is related to previous papers that have estimated a relationship between weather and the area harvested. Cui [18] estimates the impact of weather on crop abandonment of U.S. corn and soybeans using county-level panel data. Stuecker et al. [20] estimate pairwise correlations between climate variables and rice production, whereas we use modern econometric methods with panel data to estimate how yield and harvested area responds to random weather shocks. Our work also improves upon [21], where crop failure was explained as a function of weather and soil variables. A key difference is that we include location fixed effects to control for time-invariant unobserved heterogeneity that is correlated with weather. Our estimation is different from [5, 8] who only estimate the impact on yields and do not explore the effect on the proportion of acres harvested. Tack et al. [5] estimated warming impacts using wheat yield data from experimental plots in Kansas, whereas we use county-level farm data and estimate the impacts of climate change projections.

## Methods

### Data

**Fig 1:**
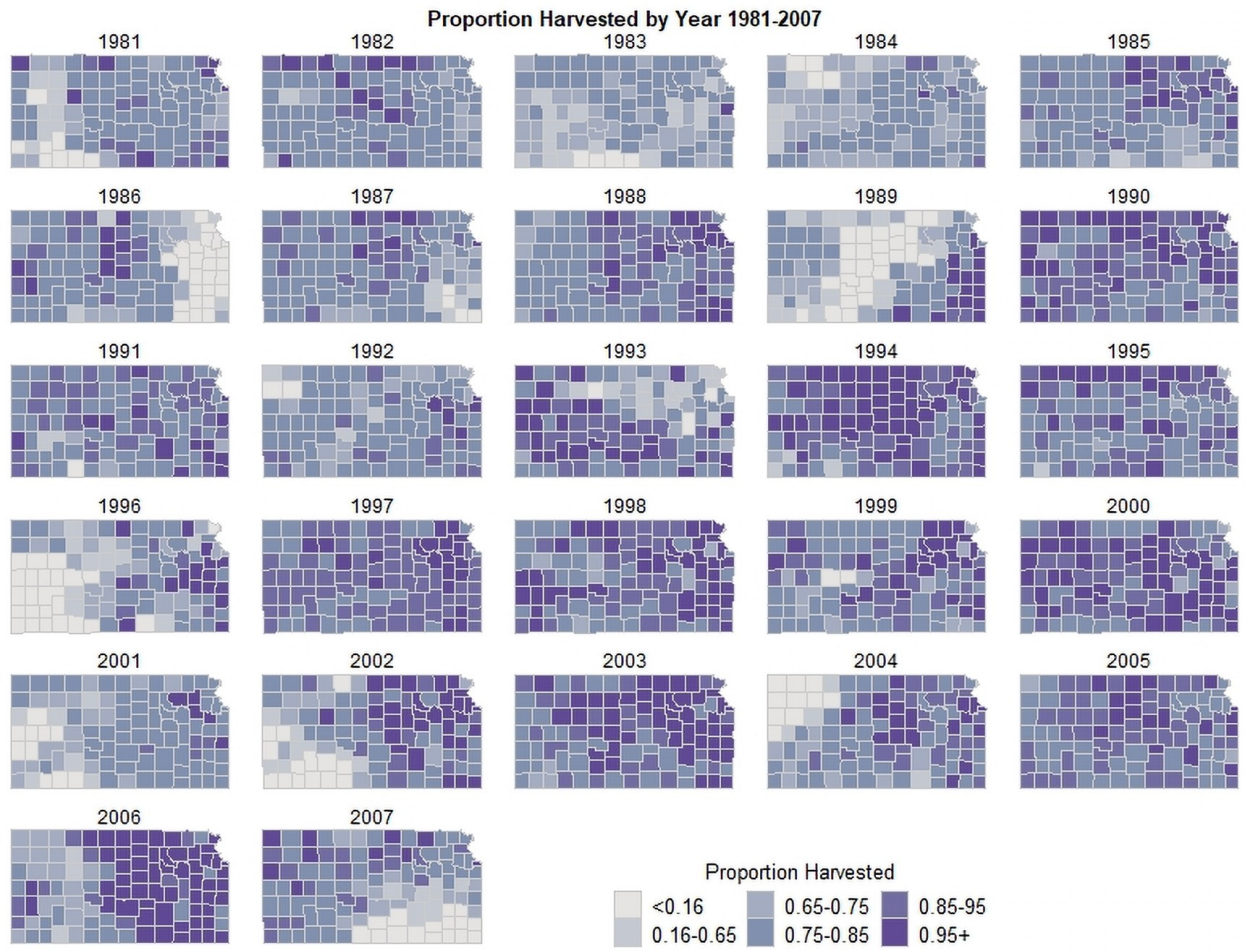
The distribution of proportion of winter wheat acres harvested in Kansas (1981–2007).

We use the USDA county-level data on production, planted, and harvested acres for dryland winter wheat in Kansas from 1981 to 2007. Several counties have missing data after 2007 due to a change in the USDA survey methodology. Fig 1 illustrates the data on proportion of acres harvested. Crop abandonment for winter wheat is as small as 4% of the total wheat area in some years, while in other years the loss from crop abandonment is as high as 25% of the total area planted. We refer to yield as the harvested yield (i.e., total production divided by harvested acres). S1 Table in S1 Appendix shows the summary statistics for the production variables. The average harvested yield between 1981–2007 is 954.01 kg/ha, while 89% of the planted acres are harvested on average.

We use the daily weather data from Parameter-elevation Relationships on Independent Slopes Model (PRISM) to construct the weather variables for the growing season duration from September to May. Following [5], we divide the growing season into three periods; Fall (September–November), Winter (December–February), and Spring (March-May). We exclude the weather information during the final parts of the growing season because the harvest in Kansas typically starts in June. S1 Table in S1 Appendix has the descriptive statistics for the weather during each of the growing periods. More details on how the degree days and freeze variables are constructed are explained in detail in S2 Section in S1 Appendix.

### Econometric model

Based on our conceptual framework (S1 Section in S1 Appendix), we model the total production response as the sum of climate effects on harvested yield and proportion harvested. First, we estimate the yield model by using a similar approach as [5] to estimate the nonlinear effect of weather variables on winter wheat yield. The yield model is specified as

$$\begin{array}{c} y_{it}=\sum_{s=1}^{3}X_{ist}\beta_{s}+\delta_{i}+\tau \left(t\right)+\varepsilon_{it}, \end{array}$$ (1)

where *y*_*it*_ is the log of winter wheat yield to remove the skewness in yield distribution across counties, *δ*_*i*_ is the county fixed effect used to control for time-invariant heterogeneity like soil quality, and *τ*(*t*) is a linear and quadratic time trend to capture changes in technology and management practices. The weather variables are denoted as a vector **X**_*ist*_ that includes degree days, exposure to freezing temperature, and precipitation. We applied time separability by allowing weather variables to have varying impacts across the growing periods denoted with the subscript *s*, where *s* = 1, 2, and 3 denote fall, winter, and spring, respectively. Standard errors are clustered by year to allow the error term to have any form of spatial correlation within a year.

The weather portion of the equation ($\sum_{s=1}^{3}X_{ist}\beta_{s}$) is defined as

$$\begin{array}{c} \begin{array}{c} \sum_{s=1}^{3}X_{ist}\beta_{s}=\sum_{s=1}^{3}\beta_{1s}Freeze_{ist}+\sum_{s=1}^{3}\beta_{2s}DDLow_{ist}\\ +\sum_{s=1}^{3}\beta_{3s}DDMedium_{ist}+\sum_{s=1}^{3}\beta_{4s}DDHigh_{ist}\\ +\sum_{s=1}^{3}\beta_{5s}Prec_{ist}+\sum_{s=1}^{3}\beta_{6s}Prec_{ist}^{2}. \end{array} \end{array}$$ (2)

We construct *Freeze*_*ist*_ as the exposure to freezing temperature in days. We construct the degree days using a sinusoidal interpolation of minimum and maximum temperature exposure within each day [5, 10]. Following [5], we estimate a piecewise linear regression using Eq (1) over different possible thresholds within each period. We define *DDLow*_*ist*_ for each period as the degree days between zero and the lower threshold, *DDMedium*_*ist*_ as the degree days between the lower and upper threshold, and *DDHigh*_*ist*_ as the degree days above the upper threshold. We use a data driven approach to estimate the temperature thresholds as in previous studies [5, 10]. We use the same principle as [5] by restricting the lower threshold at least five degrees above zero and ten degrees below the maximum observed temperature, while the upper threshold is restricted to be five degrees above the lower threshold and five degrees below the maximum for fall and spring. We then select the optimal thresholds from the model with the best fit based on *R*^2^. The descriptive statistics of the selected thresholds used in the model are in S3 Table in S1 Appendix.

The beneficial temperatures within each period vary across the growing season and appear consistent with [22] regarding the effects of temperature exposure on wheat development. Different temperature exposure ranges are needed for optimal winter wheat production [22, 23]. [24] explains that winter wheat is mostly planted when the daily temperature is between 8–16°C while an optimal temperature ranging between 12–15°C is needed for germination [23]. Temperature between 3–10°C is needed during winter for vernalization [25, 26], and a temperature below zero is damaging for yield in the spring [27]. The same thresholds are used both in the yield and proportion of acres harvested models.

Next, we describe the model for the proportion of acres harvested. We denote harvested acres and planted acres as $Acres_{it}^{H}$ and $Acres_{it}^{P}$. We estimate a linear probability model (LPM) with county fixed effects as our preferred specification. We use the LPM instead of the fractional probit model as the LPM allows the use of a wild cluster bootstrap for warming and climate change projections as described later in the paper. The wild cluster bootstrap requires additively separable errors, so it cannot be applied to the fractional probit [28]. The fixed effects LPM that we estimate is written as

$$\begin{array}{c} \frac{Acres_{it}^{H}}{Acres_{it}^{P}}=\sum_{s=1}^{3}X_{ist}\beta_{s}+\delta_{i}+\tau \left(t\right)+\eta_{it}. \end{array}$$ (3)

As a robustness check, we compare our marginal effects from the fixed effects LPM to the average partial effects from a correlated random effects fractional probit model. The correlated random effects fractional probit model allow us to avoid problems related to the incidental parameters problem that results from the inclusion of fixed effects in a nonlinear model [29] while also reducing concerns about unobserved cross-sectional heterogeneity that may bias our coefficient estimates [29–31]. Our correlated random effects model is written as

$$\begin{array}{c} \begin{array}{c} E(\frac{Acres_{it}^{H}}{Acres_{it}^{P}})=\Phi (\sum_{s=1}^{3}X_{ist}\beta_{s}+\sum_{s=1}^{3}{\bar{X}}_{is}\rho_{s}+Z_{i}\theta +\tau \left(t\right)), \end{array} \end{array}$$ (4)

where Φ(⋅) denotes the cumulative normal distribution, ${\bar{X}}_{is}=\frac{1}{T}\sum_{t=1}^{T}X_{ist}$ is a vector of average weather characteristic for county *i* and **Z**_*i*_ is a vector of soil characteristics. We assume that the unobserved factors that are uncorrelated with average weather (i.e., ${\bar{X}}_{is}$) are also independent of weather shocks (i.e., **X**_*ist*_), so that we can consistently estimate *β*_*s*_ from Eq (4), and the respective average partial effects (APEs) [29, 31]. Unobserved factors that are correlated with mean climate are controlled for in the specification by including average weather as right-hand side variables. Note that the coefficients on the average weather variables (i.e., ${\bar{X}}_{i}$) are considered nuisance parameters in this framework and are not intended to be interpreted as causal.

We compare the results from Eq (3) and the average partial effects of equation (4) in S3 Table in S1 Appendix. The average partial effects from the fractional probit (column 3 of S3 Table in S1 Appendix) are similar in sign and magnitude to the coefficients from the linear fixed effects model (column 1 of S4 Table in S1 Appendix). Therefore, we use the linear probability model results for the rest of the paper.

We project the climate change impacts on winter wheat production by mid-century (2034–2065) using our preferred econometric models with 18 different climate models under RCPs 4.5 and 8.5 with 2007 technology. We use the downscaled Coupled Model Intercomparison Project (CMIP5) daily climate projections. We make a bias correction by using the mean difference between variables constructed from historically simulated data by the climate models and the observed data from the PRISM to adjust the future data projection [32]. The list of the 18 climate models and institutions associated with the models is listed in S5 Table in S1 Appendix. We use historical data between 1981–2005 for the bias correction.

We estimate the relative changes by mid-century compared to the average historical climate by calculating the relative change in production through a change in log yield and proportion harvested. We estimate the relative change in yield as a change in the log of yield. We predict the proportion harvested and yield for each year by mid-century, decompose the drivers of changes, and quantify uncertainties from both the regression and climate models. Following [33], we use a wild bootstrap which preserves the regressors but resamples the dependent variable using the ordinary least square prediction and the residual with probability 0.5 and the negative of the residual with probability 0.5. The wild bootstrap is clustered by year to account for spatial correlation in the errors for a given year. The wild cluster bootstrap is preferred to a pairs bootstrap as a pairs cluster bootstrap can give biased estimates of standard errors with a small number of clusters [34]. We quantify the uncertainty from both the regression models and the climate models by combining the estimates from bootstrap replications across each climate model for a total of 18,000 replications for production, proportion harvested, and yield under each RCP scenario.

In order to assess the validity of the yield model, we compare results from a uniform warming impact with [5] and compare results from climate projections with [35] and the results are similar–details are provided in S2 Section in S1 Appendix.

## Results

### Freezing temperatures in the fall and extreme heat in the spring are the major drivers of yield loss

One of the drivers of yield loss is exposure to freezing temperatures during fall. An additional day of freezing in the fall reduces harvested yield by 3.54% (S4 Table in S1 Appendix). The results also show that another degree day of extreme heat in the spring decreases harvested yield by 6.08%. High temperatures in the fall have an insignificant impact on yield. We also find that an additional degree day above 11°*C* during the winter decreases yield by 0.73%.

Precipitation has a statistically significant inverted-U shape in all the seasons. A 1cm reduction in precipitation from average decreases yield by 1.35% in the fall, 1.11% in the winter, and 0.3% in the spring.

### Freezing temperatures and extreme heat during spring are the major drivers of crop abandonment

The major drivers of the proportion of acres harvested are freeze in the spring and extreme heat in the spring. An additional day of freezing temperatures in spring is associated with a 1.10 percentage point reduction in the proportion of acres harvested (column 1 of S3 Table in S1 Appendix). Intuitively, the proportion of harvested acres may increase in much colder regions as the warming impacts from the temperature increase reduce damages from freezing. Warming is more likely to have a negative impact where the increase in extreme heat will be especially large.

The proportion harvested is also highly sensitive to increased exposure to a temperature above 31°*C*. An additional degree day above 31°*C* reduces the proportion harvested by 2.33 percentage points. The effect of precipitation during the fall period is statistically significant. A 1 cm increase in precipitation increases the proportion of acres harvested by 0.4 percentage points.

### Climate change is projected to lead to large losses in wheat production

**Fig 2:**
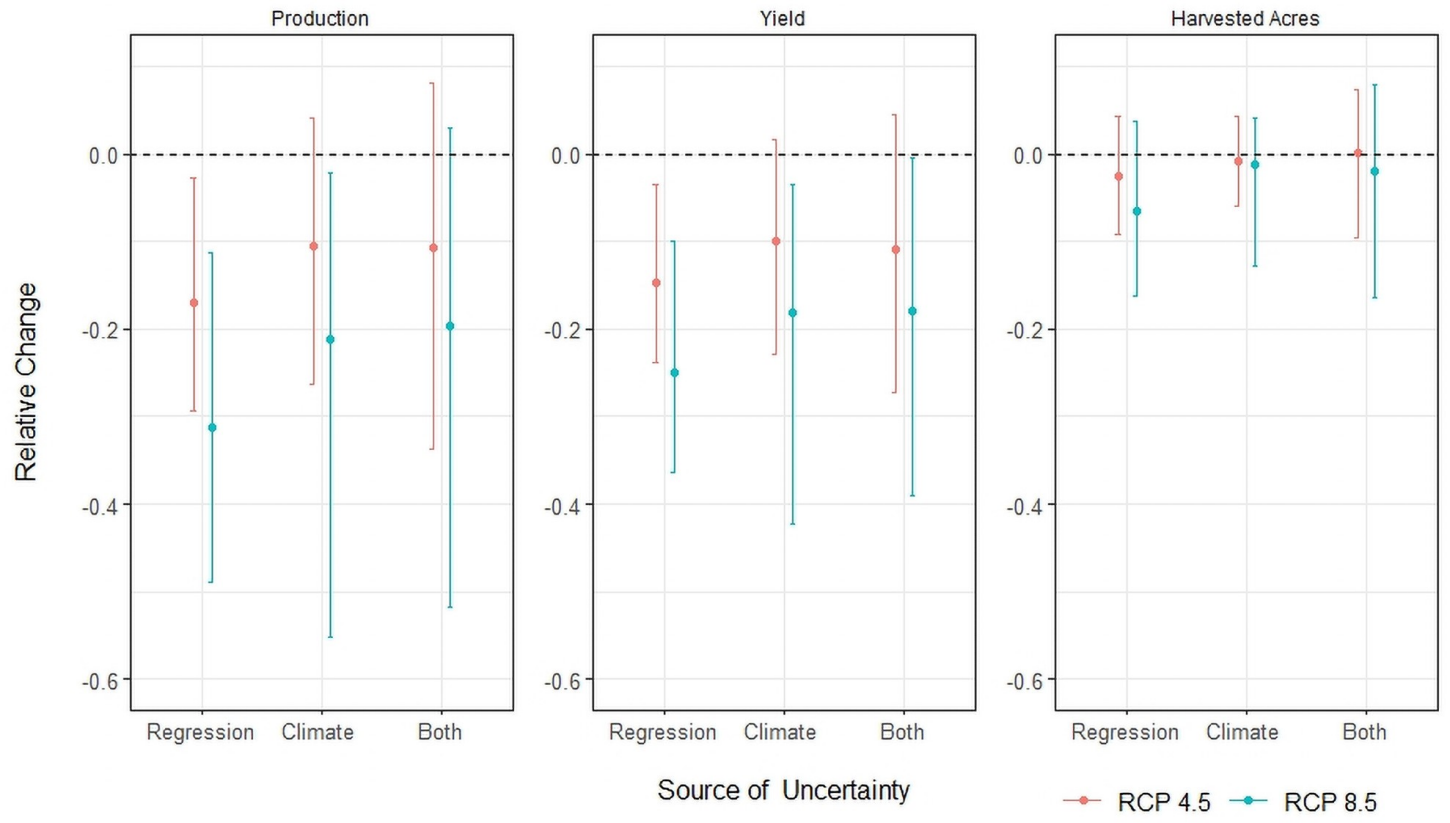
Decomposition of the relative change in production by yield and harvested acres by mid-century (2034–65) using ensemble average climate variables under RCP 4.5 and 8.5. The dot indicates the mean effect and whiskers show the 95% confidence interval. The left plot shows the total impact from yield and harvested acres, while the other two panels show the impact of yield and harvested acres. Regression uncertainty accounts for uncertainty in the statistical model, climate uncertainty accounts for uncertainty across 18 different climate models, and both combines the uncertainty from both sources.

Fig 2 shows the decomposition of the total impact of climate change on production through proportion harvested and yield response by mid-century using the decomposition described (S1 Section in S1 Appendix). In total, winter wheat production is expected to decrease by 16.96% and 31.33% by mid-century under RCPs 4.5 and 8.5. About 14.88% and 21.71% of the projected reduction in production in RCPs 4.5 and 8.5 are due to a reduction in the proportion of acres harvested, and neither is significant. The 95% confidence intervals for total production from both climate and regression uncertainty are [-33.77%, 8.03%] under RCP 4.5 and [-51.81%, 2.91%] under RCP 8.5.

Climate model uncertainty is larger than regression uncertainty for the impact of climate change on yield. The regression 95% confidence interval for the yield impact is [-23.78%, -3.44%] under RCP 4.5 and [-36.36%, -9.95%] under RCP 8.5. The most extreme impacts come from climate models MIROC5 and IPSL-CM5A-MR that project a 22.93% and 42.36% reduction in yield under RCPs 4.5 and 8.5 while climate model INMCM4 projects an increase of 1.55% in yield under RCP 4.5 and MRI-CGCM3 projects a 3.55% reduction in yield under RCP 8.5 (S3 Fig in S1 Appendix).

The regression model 95% confidence interval for the impact on the proportion of acres harvested is [-9.12%, 4.30%] under RCP 4.5. Both confidence intervals from the regression model under RCPs 4.5 and 8.5 include zero, therefore, the impact from the proportion of acres harvested is not statistically significant. For the climate models, ACCESS1–0 and IPSL-CM5A-MR models predict the largest reduction in the proportion of acres harvested at 5.91% and 12.75% under RCPs 4.5 and 8.5, while climate models CNRM-CM5 and MIROC-ESM project an increase of 4.37% and 4.05% under RCPs 4.5 and 8.5 (S4 Fig in S1 Appendix). Similar to the yield results, climate model uncertainty is larger than the uncertainty from the statistical model for the impact on proportion of acres harvested (Fig 2).

**Fig 3:**
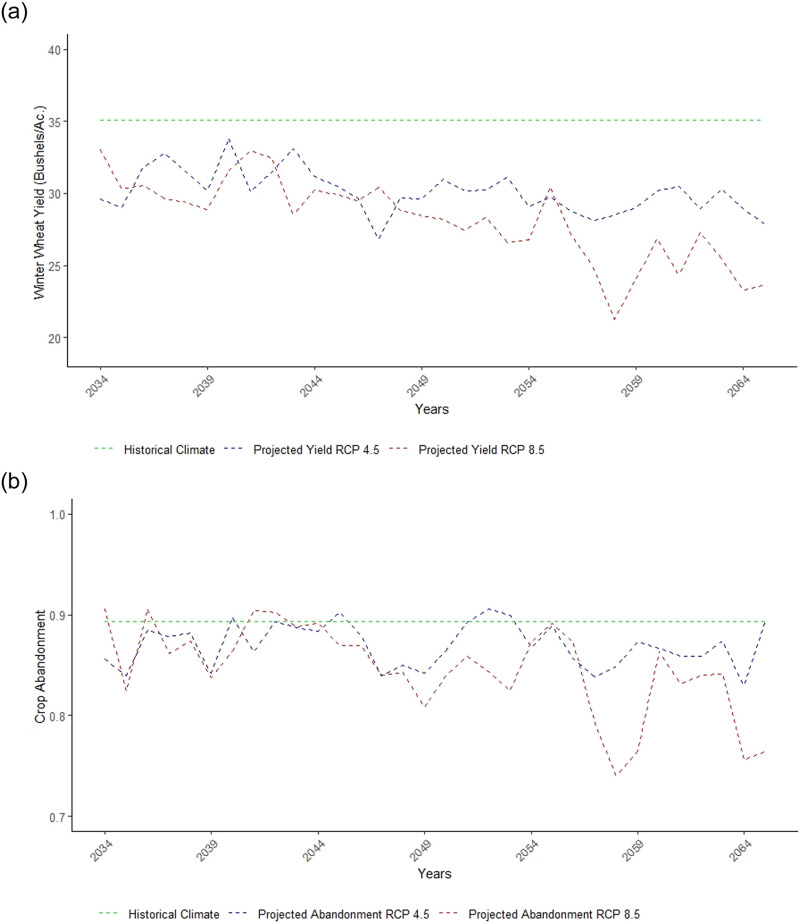
Climate change losses over time. (A) Projected yield of winter wheat by year under scenarios RCP 4.5 and RCP 8.5. The dashed green line is the average yield with a historical climate. (B) The projected proportion of acres harvested of winter wheat by year under scenarios RCP 4.5 and RCP 8.5. The dashed green line is the average proportion harvested with the historical climate.

Fig 3 shows the prediction of the ensemble average impacts on yield and proportion of acres harvested by year compared to the historical average shown by the green line. Impacts of climate change on yield get increasingly worse over time with roughly 333 Kg/ha losses in the period 2034–2050. But for RCP 8.5, yield losses increase to nearly 926 Kg/ha by 2058. Impacts on proportion harvested are mostly small until 2050. After 2050 there are larger decreases in proportion of acres harvested in RCP 8.5.

### Climatic losses are driven by extreme heat and freezing

**Fig 4:**
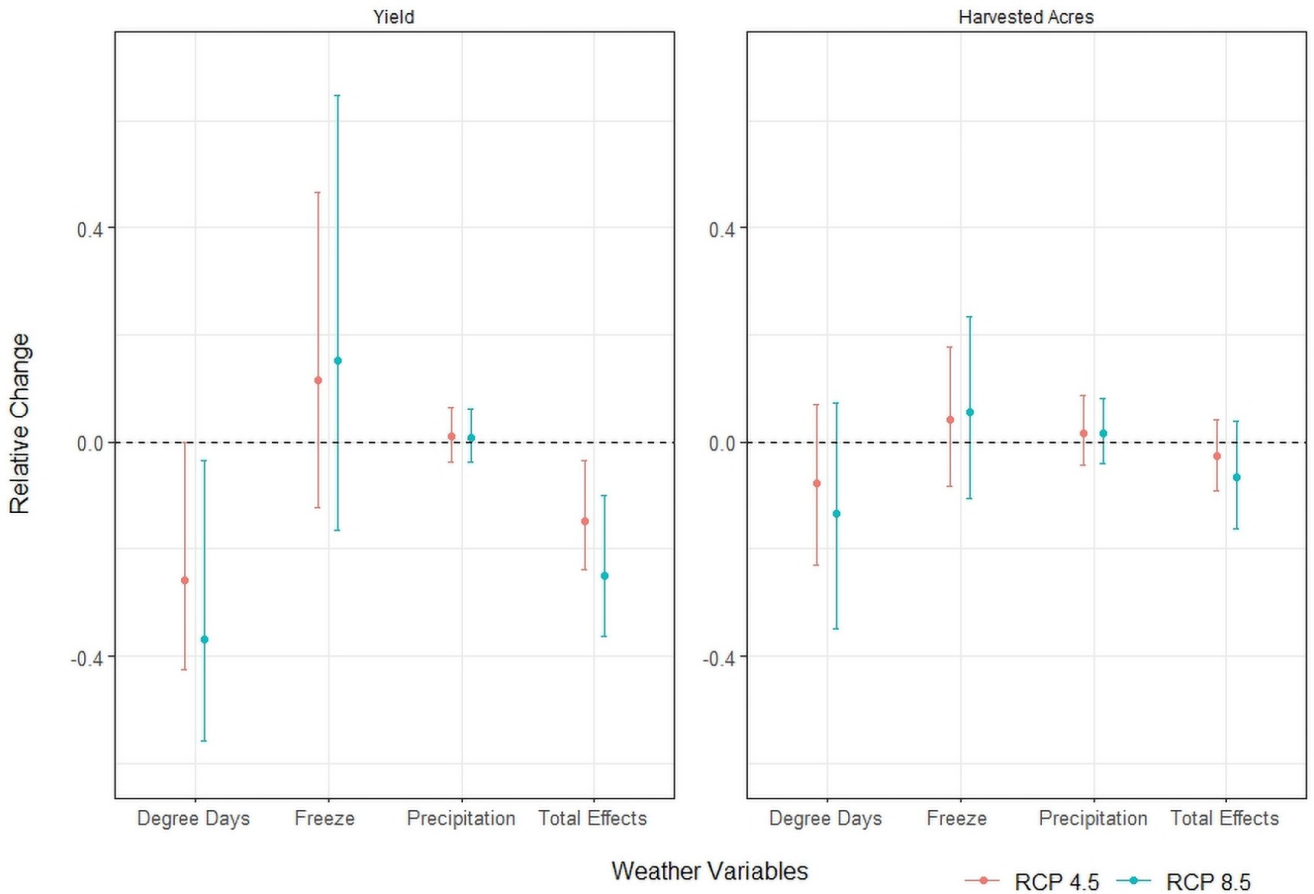
Decomposition of production losses by drivers of change. The change and in projected winter wheat yield (A) or proportion of acres harvested (B) at mid-century (2034–65) using a change in each type of climate variable. The dot indicates the mean effect and whiskers show the 95% confidence interval. Results use ensemble average climate projections and uncertainty is from the uncertainty of the regression model estimates.

Fig 4 shows the average impact on yield and proportion of acres harvested by mid-century with the ensemble average across all climate models. We decompose the total change by the potential drivers of change: (i) degree days (ii) freeze, and (iii) precipitation. To isolate the contribution of each driver, we simulate the relative change in the yield or proportion harvested by changing one driver according to projections and holding the other drivers constant.

Winter wheat yield is expected to decrease by 14.72% and 25.03% under RCPs 4.5 and 8.5, respectively (Fig 4(A)). The major driver of yield reduction by mid-century will be an increase in temperature. Using the RCP 4.5 scenario in Fig 4, an increase in temperature will decrease yield through greater degree days by 25.82%. Reduced exposure to the freezing conditions will increase winter wheat yield by 11.56%. The net impact from temperature through degree days and exposure to the freezing condition will decrease winter wheat yield by 14.26% under RCP 4.5 by mid-century. Precipitation accounts for a 0.98% increase in yield.

Using the ensemble average climate, the proportion harvested is predicted to decrease by 2.52% and 6.49% under RCPs 4.5 and 8.5 (Fig 4(B)). Under RCP 4.5, an increase in temperature through degree days alone decreases the proportion harvested by 7.71%. The reduction in damage from exposure to freezing conditions increases the proportion of acres harvested by 4.24%. The change in precipitation increases the proportion of acres harvested by 1.72%. Therefore, the temperature increase through degree days is the primary driver of the decrease in the proportion harvested, although a reduction in freezing and an increase in precipitation offsets some impacts from greater degree days.

## Discussion and conclusion

Wheat will continue to be an important staple food in the future as demand for cereals increases. An improved understanding of how climate change affects wheat production helps to develop effective adaptation strategies.

Climate change impacts agriculture through warming and drought conditions, which are likely to result in crop yield losses and reduction in the area harvested. A majority of the existing literature uses yield variability as the sole source of variation in production [8, 10, 13, 36]. Schlenker and Roberts [10] and Gammans et al. [8] estimate how weather impacts yield but assume that average growing area does not change to estimate the impact on total production. This ignores the potential relationship between the area harvested and climate change. Maunder [37] highlight that the portion of the field abandoned are probably the poorest acres that are most sensitive to climate impacts. Cui [18] shows that ignoring crop abandonment responses likely overestimates the production loss for corn but underestimates that for soybeans. Winter wheat is a long duration crop facing different temperature ranges and damages before harvest [27]. Therefore, accounting for crop abandonment could be especially important for winter wheat.

Our future projection of climate effects on winter wheat production shows a reduction between 16.96% and 31.33% for scenarios RCP 4.5 and 8.5. Although different in location, method, and projection range, Gammans et al. [8] project a yield decrease between 3.5% to 12.9% for winter wheat by 2037–2065 for France, while projections between 2050–2100 from [35] for RCPs 4.5 and 8.5 are similar to our findings. Our results show that winter wheat production would be mainly affected by the impacts of climate variables on yield as the impact on crop abandonment are small and statistically insignificant. The damage from yield represents the majority of the damage–86.23% and 91.31% of the total production damages under RCPs 4.5 and 8.5. One of the implications of this study is that research that only examines climate impacts on harvested yields are not significantly underestimating the production impacts.

Our results show that winter wheat production is susceptible to changing temperatures. Early freeze in the fall lowers the yield, while late freeze in the spring increases crop abandonment. Wheat is sensitive to freezing temperatures as cold temperatures cause injury like leaf chlorosis and death of growing points after dehardening in the spring [38]. According to [39], low temperatures injure wheat by winter killing, by which early spring freeze kills the growing point, leaf yellowing, lesions, splitting, or bending of lower stem, and late spring freeze causes sterility of the heads. The impact of freezing from our result in the fall is consistent but smaller in magnitude than the 9% reduction in yield estimated by [5]. The reason for the different results is likely due to differences in the type of data—we use county-level farm yields and [5] use plot-level experiment station yields. Gammans et al. [8] also find exposure to a temperature below -6°C in the fall reduces wheat yield. Barlow et al. [40] identified freeze to have the most significant impacts on production as it is associated with sterility and the abortion of formed grains around anthesis. Late frost during early grain filling causes yield loss between 13 and 33% as grains were 80% lighter after spikes [41].

Our results indicate that an increase in temperature during the winter and spring lowers yield and increases crop abandonment. According to [39], high temperatures during the winter can stimulate wheat to grow so that the subsequent low temperatures in the spring will injure the crop. Li et al. [42] find warm temperatures during the winter stop hardening early, setting the crop for further damage when cold temperatures set in during spring. Extreme heat damage is most common during grain filling when the kernels are shriveled and prematurely ripe [39]. High temperature hastens the decline in photosynthesis and leaf area, decreases shoot and grain mass, weight and sugar content of kernels, and reduces water-use efficiency [43]. O’Leary et al. [44] shows that temperature impacts were more significant at warmer temperatures than at colder temperatures. Increased heat stress causes crop failure resulting in leaf senescence [45], and the shorting of the grain filling period [36]. Lobell et al. [46] found a temperature above 34°C to accelerate wheat senescence grown in northern India. This implies that the effects of temperature change would be spatially different across locations due to variations in soil characteristics and weather conditions across counties. Crop abandonment could be reduced in the colder regions as the warming impacts from the temperature increase offset the cold condition’s damage by mid-century. The climate change impacts are different for counties in the warmer regions as an increase in temperature aggravates the hot conditions.

Precipitation is also important for winter wheat production. Wheat planted under severe drought has the shortest grain filling duration [47], lowering productivity or resulting in crop failure. According to [48], drought affects winter wheat yield significantly during the flowering and filling stages, with droughts of higher intensity having more significant negative effects on the winter wheat yield. Drought also decreases wheat quality, which may lead many farmers to not harvest [49]. Tack et al. [50] highlights the importance of irrigation in limiting the effects of drought and heat stress on winter wheat.

The primary driver of production loss due to climate change will be heat stress. The impact from temperature increases on extreme heat outweigh the benefits from reduced freezing conditions and greater precipitation. The impacts from extreme heat can be mitigated through the development of heat stress resistant winter wheat varieties [5].

There are a few limitations to our work worth recognizing. We project the impacts of climate change, assuming no adaptation. One way to interpret our results is that they illustrate the potential value from adaptation. For example, the development and adoption of heat-tolerant winter wheat varieties could mitigate losses. Our analysis does not consider the fertilization effects of *CO*_2_ on winter wheat production [51]. Winter wheat water use efficiency is higher under a high level of *CO*_2_ [52]. Another limitation is that we only analyze winter wheat production using data from Kansas—the impacts could be more significant in other production regions of the world, especially in the tropics [19]. While we find that crop abandonment represents a small proportion of climatic damages for winter wheat, the impacts could be different for other crops.

Crop abandonment is especially large for winter wheat in Kansas, so this is a setting where we would expect to see one of the largest impacts of weather on crop abandonment. But given the fact that the impact found was small in Kansas, we expect similar results for the rest of the USA. Future work could see if these results extend to other crops and other regions of the world and the role of crop insurance on crop abandonment. Given that much of the projected production losses are from extreme heat, it is important for future research to investigate different ways to mitigate the impact of heat losses.

## Supporting information

S1 Appendix (PDF)

S1 Data (DTA)

S1 Fig (TIF)
