# Supplementary material for: Decreased wheat production in the USA from climate change driven by yield losses rather than crop abandonment
Source: PLoS One. 2021 Jun 17;16(6):e0252067. doi: 10.1371/journal.pone.0252067 (PMC8211167; doi:10.1371/journal.pone.0252067)
Supplement: S1 Appendix — (PDF) [file pone.0252067.s001.pdf]

Appendix

S1 Conceptual Framework

We define annual production ($Prod_{it}$) as a product of harvested yield and harvested area [2]. For this study, we assume cropping intensity for dryland winter wheat to be one crop per year because the growing season is sufficiently short in Kansas that double-cropping is still uncommon. We define harvested yield as production divided by area harvested while we defined planted yield as production divided by area planted. Let $y_{it}^{H}=\dfrac{Prod_{it}}{Acres^{H}}$ denote harvested yield and $y_{it}^{P}=\dfrac{Prod_{it}}{Acres^{P}}$ denote the planted yield. Let y_{it} be a function of weather variables W_{it} (i.e., temperature and precipitation) given that other variables are controlled for in the empirical analysis,

$$y_{it} = f(W_{it}). \tag{S5}$$

We denote harvested acres and planted acres as $Acres^{H}(W_{it})$ and $Acres^{P}$. We assume that planted acres do not depend on the weather, $\dfrac{\partial Acres^{P}}{\partial W_{it}} = 0$, because planting decisions are made before weather outcomes are known. Only a proportion of the total area planted is harvested due to crop failure on some acres. We express the proportion of the area harvested as $\frac{Acres^{H}(W_{it})}{Acres^{P}}$. Production can be written as

$$Prod_{ist}(W_{it}) = y_{it}^{P}(W_{it}) \times Acres^{P} = y_{it}^{H}(W_{it}) \times Acres^{H}(W_{it}). \tag{S6}$$

We can express production on a per acre basis by dividing both sides by $Acres^{P}$, since $Acres^{P}$ do not depend on weather, giving the expression

$$y_{it}^{P}(W_{it}) = y_{it}^{H}(W_{it}) \times \frac{Acres^{H}(W_{it})}{Acres^{P}}. \tag{S7}$$

Taking the derivative of equation S7 gives the impact of climate change on production,

$$y_{it}^{P'} = y_{it}^{H'} \times \left\{\frac{Acres^{H}}{Acres^{P}}\right\} + \left\{\frac{Acres^{H}}{Acres^{P}}\right\}' \times y_{it}^{H}, \tag{S8}$$

where the primes denote the first derivative with respect to weather. To express the change in production in relative terms we divide equation S8 by $y^{p} = y_{it}^{H} \times \frac{Acres^{H}}{Acres^{P}}$,

$$\frac{y_{it}^{P'}}{y_{it}^{p}} = \frac{y_{it}^{H'}}{y_{it}^{H}} + \frac{\left\{\frac{Acres^{H}}{Acres^{P}}\right\}'}{\left\{\frac{Acres^{H}}{Acres^{P}}\right\}}. \tag{S9}$$

Equation S9 shows that the relative change in total production $\left(\frac{y_{it}^{P'}}{y_{it}^{p}}\right)$ is equal to the relative change in harvested yield $\left(\frac{y_{it}^{H'}}{y_{it}^{H}}\right)$ plus the relative change in acres harvested $\left(\frac{\left\{\frac{Acres^{H}}{Acres^{P}}\right\}'}{\left\{\frac{Acres^{H}}{Acres^{P}}\right\}}\right)$. Note that the relative change in the proportion of area harvest is equal to the percent change in harvested area, $\frac{\left\{\frac{Acres^{H}}{Acres^{P}}\right\}'}{\left\{\frac{Acres^{H}}{Acres^{P}}\right\}} = \frac{Acres^{H'}}{Acres^{H}}$ because $Acres^{P}$ are not a function of weather. We write the model in terms of the proportion of area harvested to correspond with our econometric model. If weather has a negative impact on the proportion of area harvested, then models that only estimate the impact on harvested yield will understate the impact of climate change on total production.

S2 Data

S1 Table. Descriptive Statistics of Production, Weather, and Soils Measures

Production Measures		Mean	Std. Dev.	Min	Max
Harvested Yield(Kg/ha)		954.01	275.55	244.94	2186.64
Harvested Area (1,000 ha)		36.67	27.58	0.08	204.61
Planted Area (1,000 ha)		41.11	29.88	0.08	212.06
Proportion Harvested		0.89	0.12	0.00	1
Production (1,000 Kg)		87,711.33	72,303.61	163.32	597,786.6
Weather measures	Seasons				
	Fall	13.30	1.44	8.53	17.47
Temperature (^oC)	Winter	0.33	1.91	-5.79	5.28
	Spring	12.27	1.58	6.92	15.82
	Fall	167.67	99.06	15.59	748.87
Precipitation (mm)	Winter	79.86	48.81	9.70	349.87
	Spring	234.31	94.53	40.48	625.67
Soil measures					
Rootznaws (mm)		247.34	39.71	160.28	311.74
Soil organic carbon (kg/m^3)		11.51	2.94	5.63	81.89
Bulk density		1.38	0.06	1.21	1.54
Electrical conductivity (EC)		0.39	0.32	0.00	1.19
pH less than 6		0.04	0.10	0.00	0.62
pH greater than 7.5		0.44	0.41	0.00	1.00

Note: Harvested yield is calculated as production divided harvested acres. The data are from 105 counties in Kansas between 1981 and 2007. Weather variables are aggregated over the growing seasons.

S2 Table. Optimal Thresholds from Piecewise Linear Models

Variables	Threshold	Mean ± SD.
Degree Days low:Fall	0–10 ^oC	716.69 ± 50.43
Degree Days Medium:Fall	10–15 ^oC	243.39 ± 27.15
Degree Days High:Fall	15+ ^oC	277.27 ± 53.15
Degree Days low:Winter	0–5 ^oC	172.57 ± 38.12
Degree Days Medium:Winter	5–11 ^oC	95.60 ± 31.83
Degree Days High:Winter	11+ ^oC	30.78 ±18.73
Degree Days low:Spring	0–20 ^oC	1079.36 ± 112.72
Degree Days Medium:Spring	20–31 ^oC	76.17 ± 27.65
Degree Days High:Spring	31+ ^oC	1.12 ± 1.98

Note: The thresholds were estimated through piecewise regression over all possible thresholds. The optimal thresholds for the yield model were selected from the models that maximize the R^2. Seasons are defined as September-November (Fall), December-February (Winter), and March-May (Spring).

S3 Table. Econometric Results of Weather Effects on Proportion Harvested

Estimation Methods	(1) Linear Fixed Effects	(2) Fractional probit Pooled QMLE	
Variables	Coefficient	Coefficient	APE
Freeze Days: Fall	-0.0081	-0.0496	-0.0087
	(0.0083)	(0.0465)	(0.0099)
Freeze Days: Winter	-0.0015	-0.0123	-0.0021
	(0.0068)	(0.0098)	(0.0076)
Freeze Days: Spring	-0.0110**	-0.0553**	-0.0097*
	(0.0053)	(0.0259)	(0.0064)
Degree Days low: Fall	-0.0001	-0.0009	-0.0001
	(0.0011)	(0.0055)	(0.0012)
Degree Days Medium: Fall	-0.0004	-0.0012	-0.0002
	(0.0015)	(0.0080)	(0.0016)
Degree Days High: Fall	0.0001	-0.0007	-0.0001
	(0.0003)	(0.0017)	(0.0004)
Degree Days low: Winter	-0.0001	-0.0001	-0.0000
	(0.018)	(0.0099)	(0.0021)
Degree Days Medium: Winter	-0.0003	-0.0020	0.0004
	(0.0015)	(0.0078)	(0.0016)
Degree Days High: Winter	0.0006	0.0041	-0.0007
	(0.0013)	(0.0061)	(0.0013)
Degree Days low: Spring	-0.0004	-0.0021	0.0004
	(0.0003)	(0.0014)	(0.0004)
Degree Days Medium: Spring	0.0012	0.0060*	-0.0011
	(0.0007)	(0.0037)	(0.0008)
Degree Days High: Spring	-0.0233**	-0.1048***	-0.0183**
	(0.0077)	(0.0328)	(0.0080)
Precipitation(mm): Fall	0.0009**	0.0044**	0.0008
	(0.0004)	(0.0018)	(0.0005)
Precipitation(mm) Squared: Fall	-0.0000**	-0.0000***	-0.0000
	(0.0000)	(0.0000)	(0.0000)
Precipitation(mm): Winter	0.0002	0.0011	0.0002
	(0.0005)	(0.0019)	(0.0001)
Precipitation(mm) Squared: Winter	-0.0000	-0.000	0.0000
	(0.0000)	(0.0000)	(0.0000)
Precipitation(mm): Spring	0.0004	0.0021	0.0004
	(0.0004)	(0.0027)	(0.0004)
Precipitation(mm) Squared: Spring	-0.0000	-0.0000	0.0000
	(0.0000)	(0.0000)	(0.0000)
Average Weather Controls	No		Yes
Soil Controls	No		Yes
County Fixed effect	Yes		No
Quadratic Trend	Yes		Yes

Note: *,** and *** indicate significance at 0.1 and 0.05 and 0.01 levels. Numbers in the parenthesis are standard errors clustered by year. The standard errors for the APEs are obtained from 500 bootstrap replications.

Estimation Results

S4 Table. Econometric Result of Weather Effects on Yield

Variables	(1) Coefficient
Freeze Days : Fall	-0.0354*
	(0.0205)
Freeze Days: Winter	-0.0026
	(0.0038)
Freeze Days: Spring	-0.0152
	(0.0152)
Degree Days low: Fall	-0.0047*
	(0.0024)
Degree Days Medium: Fall	0.0039
	(0.033)
Degree Days High: Fall	-0.0001
	(0.0007)
Degree Days low: Winter	-0.0030*
	(0.0056)
Degree Days Medium: Winter	0.0067
	(0.0042)
Degree Days High: Winter	-0.0073*
	(0.037)
Degree Days low: Spring	-0.0014*
	(0.0008)
Degree Days Medium: Spring	0.0031**
	(0.0015)
Degree Days High: Spring	-0.0608***
	(0.0159)
Precipitation(mm): Fall	0.0025***
	(0.0007)
Precipitation(mm) Squared: Fall	-0.0000***
	(0.0000)
Precipitation(mm): Winter	0.0032**
	(0.0013)
Precipitation(mm) Squared: Winter	-0.0000***
	(0.0000)
Precipitation(mm): Spring	0.0032**
	(0.0012)
Precipitation(mm) Squared: Spring	-0.0000***
	(0.0000)
County Fixed effect	Yes
Quadratic Trend	Yes

Note: *,** and *** indicate significance at 0.1 and 0.05 and 0.01 levels. Numbers in the parenthesis are standard errors clustered by year.

Validation of Yield Model with Other Models

In order to compare our result with [6], we estimate a warming scenario using equations (1) and (3) to simulate the impact of an increase in temperature on yield and proportion harvested for each 1^oC increase in temperature up to 5^oC. The impact on yield is specified as the change in winter wheat yield due to an increase in temperature compared to the yield from the historical climate while holding precipitation constant. Similarly, we simulate the impact of an increase in temperature on the proportion of acres harvested. A 2^oC increase in temperature reduces production by 15.09%, yield by 15.19% and increases the proportion of acres harvested by less than 0.1% (S1 Fig). At 2^oC, the warming impacts from the degree days far outweigh the benefits from the reduced exposure to freezing temperatures (S2 Fig). Our predicted yield reduction under 1^oC is similar in magnitude with both US and global aggregate impacts predicted by [1, 3, 5] while our estimate at 2^oC is similar to the impact from [6].

S1 Fig. Predicted impacts of warming scenarios on production through proportion harvested and yield. The first and second bars show the predicted impact of an increase in temperature across different periods during the growing season on yield and proportion harvested respectively. The third bar shows the total effects of temperature increase on production. Impacts are reported as the percentage change in yield and proportion harvested relative to the historical average values. Bars show 95% confidence intervals using standard error clustered by year and obtained from 1000 wild bootstraps.

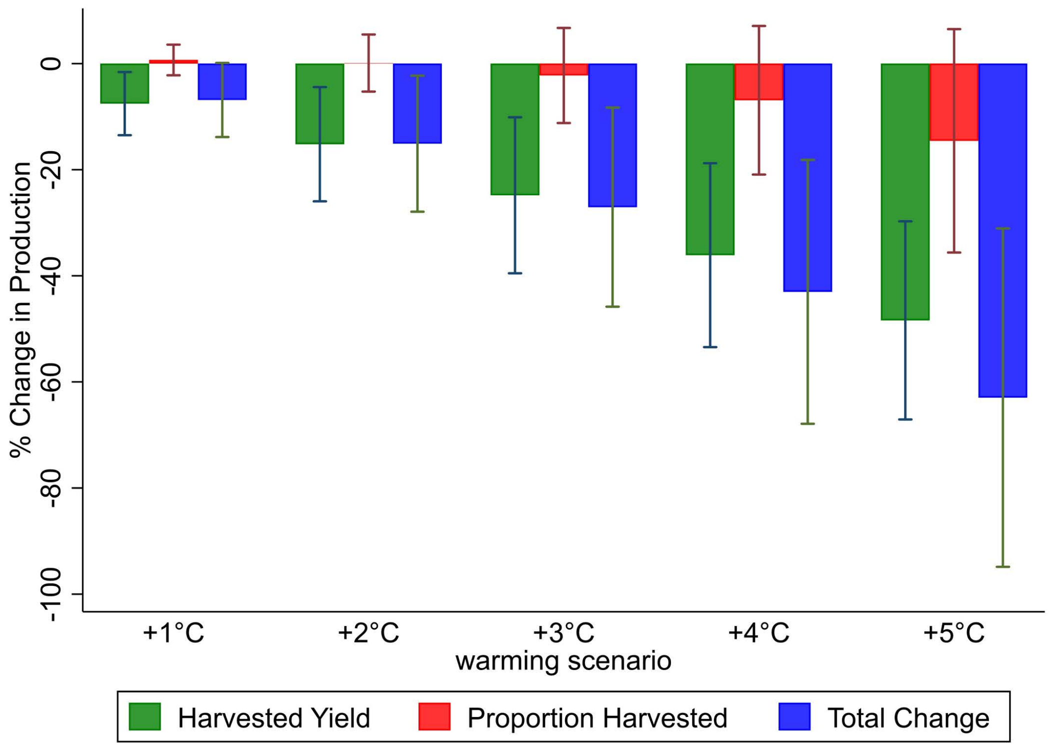

S2 Fig. This figure shows the breakdown of S1 Fig under 2^oC. The predicted impacts show the source of change through temperature effects on production. The first and second bars show the predicted impact of an increase in temperature across different periods during the growing season on yield and proportion harvested respectively. The third bar shows the total effects of temperature increase on production. Impacts are reported as the percentage change in predicted yield and proportion harvested relative to historical climate values. Bars show 95% confidence intervals using standard error clustered by year and obtained from 1000 wild bootstraps.

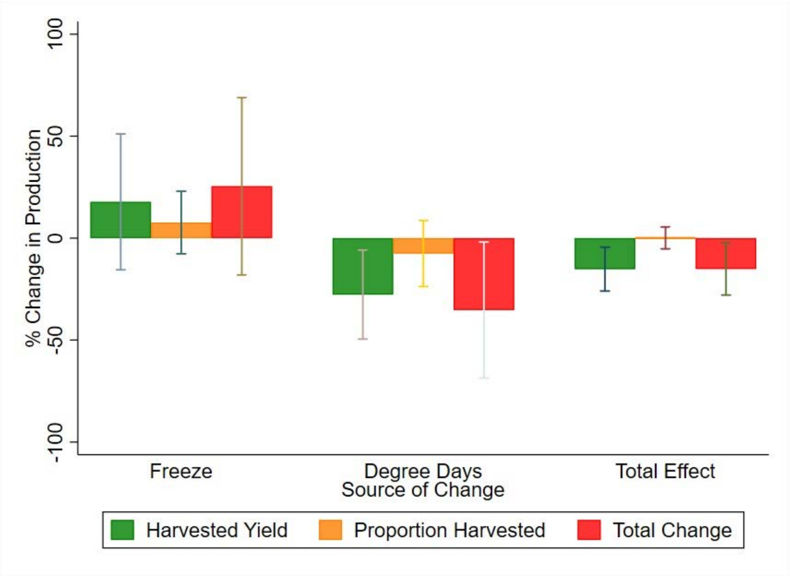

We also compare our climate projection results with findings from [4]. Although our projection timeline, the type and number of climatic models and Representative Concentration pathways (RCP) and the study area are different, our results for the climate change projection was similar to results from [4] for RCPs 4.5 and 8.5. Our result shows that winter wheat yield is expected to decrease by 14.72% under RCP 4.5 and 25.03% under RCP 8.5 by 2034-2065, respectively. [4] result for 2050-2100 shows that winter wheat is expected to decrease by 12.5% for RCP 4.5 and 20.1% for RCP 8.5.

Additional Climate Change Results

S3 Fig. Percent change in winter wheat yield across 18 general circulation models under RCP 4.5 and 8.5, respectively.

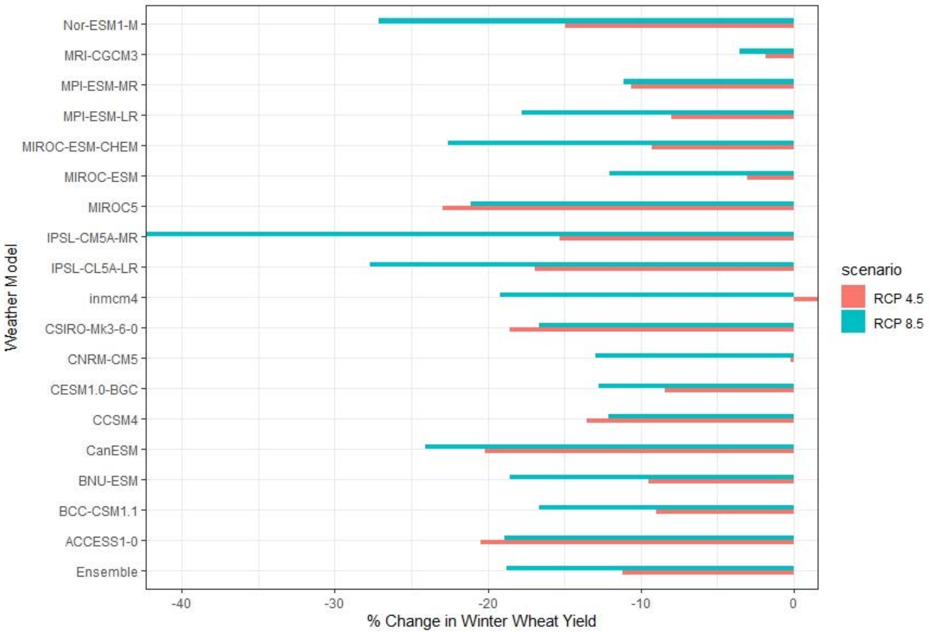

S4 Fig. Percent change in proportion of acres harvested across 18 general circulation models under RCP 4.5 and 8.5, respectively.

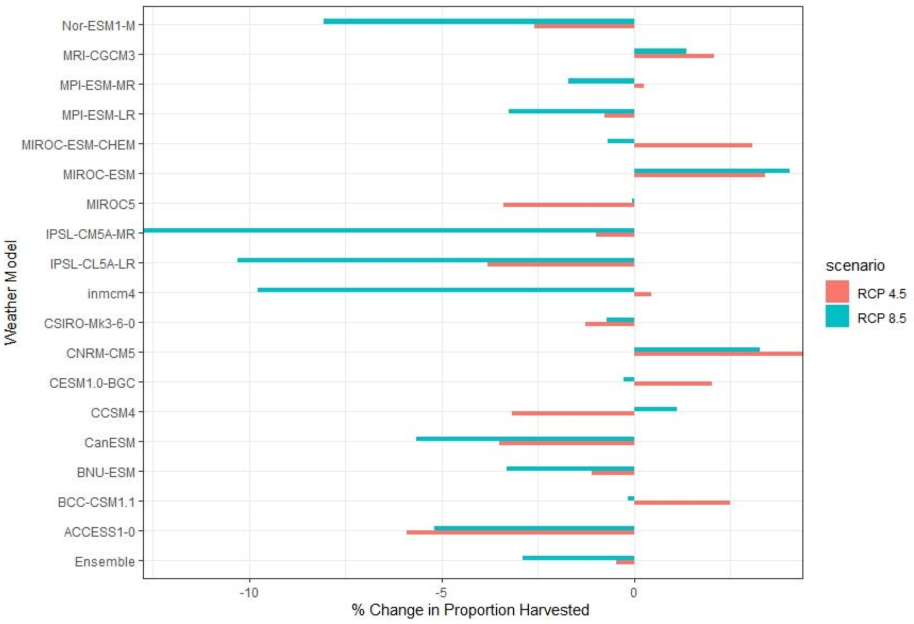

S5 Table. List of global climate models and their source

Models	Sources
ACCESS 1.0	Common Wealth Scientific and Industrial Research Organization and Bureau of Meteorology, Australia
BCC-CSM1.1	Beijing Climate Center, China Meteorological Administration, China
BNU-ESM	Beijing Normal University, China
CanESM	Canadian Center for climate modeling and analysis, Canada
CCSM4	National Center for Atmospheric Research (NCAR), USA
CESM1.0-BGC	National Center for Atmospheric Research (NCAR), USA
CNRM-CM5	Centre National de Recherches Météorologiques, France
CISRO-MK3	Australian Common Wealth Scientific and Industrial Research Organization
inmcm4	Institute of Numerical Mathematics, Russian Academy of Sciences
IPSL-CL5A-LR	Institute Pierre-Simon Laplace, France
IPSL-CL5A-MR	Institute Pierre-Simon Laplace, France
MIROC5	Japan Agency for Marin-Earth Science and Technology, Atmosphere and Ocean Research Institute
MIROC-ESM	Japan Agency for Marin-Earth Science and Technology, Atmosphere and Ocean Research Institute
MIROC-ESM-CHEM	Japan Agency for Marin-Earth Science and Technology, Atmosphere and Ocean Research Institute
MPI-ESM-LR	Max Plank Institute for Meteorology (MPI-M), Germany
MPI-ESM-MR	Max Plank Institute for Meteorology (MPI-M), Germany
MRI-CGCM3	Meteorological Research Institute of Japan
Nor-ESM1-M	Norwegian Climate Center, Norway

References

[1] Senthold Asseng et al. “Rising Temperatures Reduce Global Wheat Production”. In: *Nature Climate Change* 5.2 (2015), p. 143.

[2] Toshichika Iizumi and Navin Ramankutty. “How Do Weather and Climate Influence Cropping Area and Intensity?” In: *Global Food Security* 4 (2015), pp. 46–50.

[3] Bing Liu et al. “Similar Estimates of Temperature Impacts on Global Wheat Yield by Three Independent Methods”. In: *Nature Climate Change* 6.12 (2016), p. 1130.

[4] Ariel Ortiz-Bobea, Haoying Wang, Carlos M Carrillo, and Toby R Ault. “Unpacking the climatic drivers of US agricultural yields”. In: *Environmental Research Letters* 14.6 (2019), p. 064003.

[5] Jesse Tack, Andrew Barkley, and Nathan Hendricks. “Irrigation Offsets Wheat Yield Reductions from Warming Temperatures”. In: *Environmental Research Letters* 12.11 (2017), p. 114027.

[6] Jesse Tack, Andrew Barkley, and Lawton Lanier Nalley. “Effect of Warming Temperatures on US Wheat Yields”. In: *Proceedings of the National Academy of Sciences* 112.22 (June 2015), pp. 6931–6936. ISSN: 0027-8424, 1091-6490. DOI: 10.1073/pnas.1415181112. URL: http://www.pnas.org/lookup/doi/10.1073/pnas.1415181112 (visited on 04/19/2017).
