# Supplementary figures and images for: Decreased wheat production in the USA from climate change driven by yield losses rather than crop abandonment
Source: PLoS One. 2021 Jun 17;16(6):e0252067. doi: 10.1371/journal.pone.0252067 (PMC8211167; doi:10.1371/journal.pone.0252067)

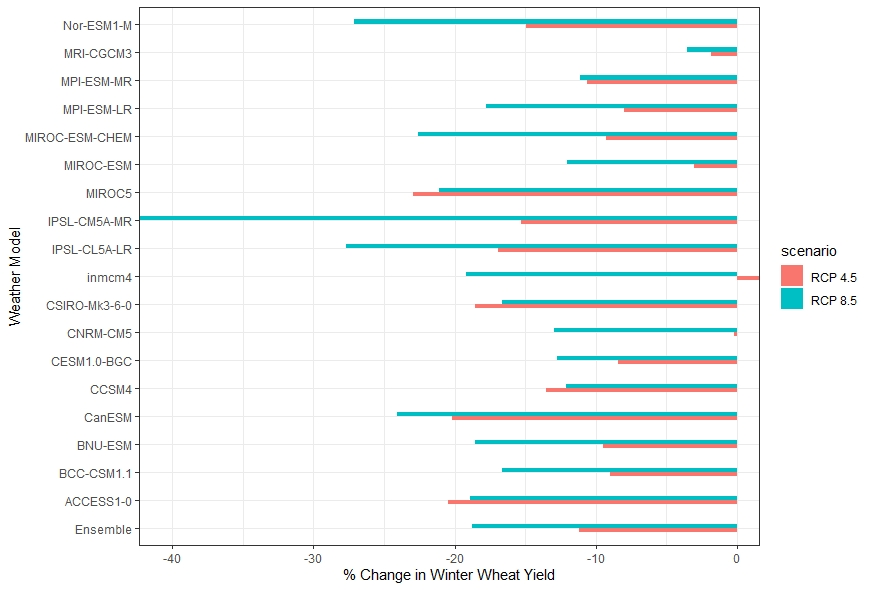

Supplement: S1 Fig — (TIF) [file pone.0252067.s003.tif]
